# ﻿*Salviapenghuana* (Lamiaceae), a new species from Guizhou, China

**DOI:** 10.3897/phytokeys.246.130332

**Published:** 2024-09-02

**Authors:** Feng-Jin Qiu, Deng-Li Yu, Hong-Bo Lan, Ming-Tai An, Yan-Fei Geng, Chun-Lei Xiang, Guo-Xiong Hu

**Affiliations:** 1 College of Life Sciences, Guizhou University, Guiyang 550025, Guizhou, China; 2 Key Laboratory of Plant Resource Conservation and Germplasm Innovation in Mountainous Region (Ministry of Education), Guizhou University, Guiyang 550025, Guizhou, China; 3 Management Department of Maolan National Nature Reserve, Libo 558400, Guizhou, China; 4 Kuankuoshui National Nature Reserve Administration, Zunyi 563300, Guizhou, China; 5 College of Forestry, Guizhou University, Guiyang 550025, Guizhou, China; 6 Key Laboratory for Plant Diversity and Biogeography of East Asia, Kunming Institute of Botany, Chinese Academy of Sciences, Kunming 650201, Yunnan, China

**Keywords:** *Salviacavaleriei*, *Salviafilicifolia*, sect. *Sobiso*, stamen movement, Subg. *Glutinaria*

## Abstract

*Salviapenghuana*, a new species from Guizhou Province of southwestern China, is described and illustrated. Morphologically, *Salviapenghuana* is similar to *S.filicifolia*, but can be easily distinguished from the latter by ovate-lanceolate bracts, purple corolla, and foot-shaped fused lower arms of connective. In addition, *S.penhuana* is morphologically similar to *S.cavaleriei*, but differs by having 3–4-pinnate leave, ovate-lanceolate bracts, puberulent calyx, and longer upper arms of connective. Based on the fibril root, small calyx and corolla, and completely reduced posterior thecae, *S.penghuana* should be placed in section Sobiso of subg. Glutinaria.

## ﻿Introduction

As currently defined, the genus *Salvia* L. includes the five traditionally defined genera (*Dorystaechas* Boiss. & Heldr. ex Benth., *Meriandra* Benth., *Perovskia* Kar., *Rosmarinus* L., and *Zhumeria* Rech. f. & Wendelbo) and is classified into 11 subgenera (Drew et al. 2017; Hu et al. 2018; Kriebel et al. 2019; Moein et al. 2023). With approximately 1000 species, *Salvia* is the largest genus within Lamiaceae (Walker et al. 2004; Wei et al. 2015; Hu et al. 2018), and has a subcosmopolitan distribution, but mainly radiates in Mesoamerica/South America, Southwestern Asia and the Mediterranean region, and Eastern Asia (Walker and Sytsma 2007; Wei et al. 2015; Hu et al. 2018). Recently, a large number of new species or hybrids of this genus have been reported around the world (Celep et al. 2020; González-Gallegos et al. 2021, 2023; Ilçim et al. 2023; Jin et al. 2023; Huang et al. 2024).

In East Asia, ca. 100 *Salvia* species have been recorded, most of which are found in China. To date, 89 native species (Li and Hedge 1994; Hu et al. 2014, 2017; Hu and Peng 2015; Chen et al. 2016; Wang et al. 2016; Xiang et al. 2016; Ding et al. 2019; Wei et al. 2019, 2021; Jin et al. 2023; Huang et al. 2024) and three naturalized species from the New World (viz. *S.coccine*a Buc’hoz ex Etl., *S.reflexa* Hornem., and *S.tiliifolia* Vahl) have been reported in China (Li and Hedge 1994; Hu et al. 2013; Shao et al. 2019). Based on the staminal morphology, Salvia in East Asia had been placed in subg. Salvia, subg. Sclarea (Moench) Benth., and subg. Allagospadonopsis Briq. (Sun and Wu 1977; Murata and Yamazaki 1993). Recently, based on molecular and morphological evidence, Hu et al. (2018, 2020) classified East Asiatic *Salvia* into two subgenera. *Salviagrandifolia* (endemic to the Hengduan Mountains) and *S.deserta* (distributed in Xinjiang of China, and Central Asia) are retained in the subg. Sclarea that includes ca. 120 species mainly from Southwestern Asia, Europe, Mediterranean region (Kriebel et al. 2019; Hu et al. 2020), and the other East Asiatic *Salvia* species were placed in the newly established subg. Glutinaria (Raf.) G.X.Hu, C.L.Xiang & B.T.Drew (Hu et al. 2018, 2020).

In January 2021, we were attracted by a *Salvia* population with 3–4-pinnately compound leaves in Libo, southern Guizhou, China. The plants without flowers morphologically resemble *S.filicifolia* Merr. Another population was later found in Kuankuoshui National Nature Reserve (northern Guizhou, China) in April 2021 and some living materials were collected and cultivated at Guizhou University. After careful observation of the flower morphology of the cultivated plants, we confirmed that these collections are not *S.filicifolia*, and may represent a new species. Over the next three years, we continued to observe the species in the field and carefully compared it with other species of *Salvia*. Finally, we confirmed that the new collections represented an undescribed species, and therefore described the new species here.

## ﻿Materials and methods

Specimens of the potential new species were collected in Libo and Suiyang counties, Guizhou Province, China. Morphological comparisons between the new species and its morphologically similar species (*S.cavaleriei* and *S.filicifolia*) were performed based on fresh materials as well as herbarium specimens deposited at GACP and KUN. Ten diagnostic characters involved in leaf, verticillaster, bract, calyx, corolla, stamen, and nutlet were selected to conduct the comparisons (Table 1). Morphological descriptions mainly referred to the Flora of China (Li and Hedge 1994).

**Table 1.** Comparison of morphological characters between *Salviapenghuana* and its morphologically similar species.

Characters	*Salviapenghuana*	*Salviafilicifolia*	*Salviacavaleriei*
Leaves	3–4-pinnate	3–4-pinnate	simple to 2-pinnate
Verticillasters	6-flowered	6–10-flowered	2–6-flowered
Bracts	ovate-lanceolate	linear-lanceolate	lanceolate
Calyx tube	sparsely glandular or puberulent along veins outside, glabrous or apically fine strigose inside	sparsely glandular or villous along veins outside, sparsely villous annulate inside	glabrous outside, apically fine strigose inside
Corolla color	purple	yellow or white	blue-purple to purple-red or white
The middle lobe of the lower lip of corolla	subrectangular	obcordate	obcordate
Lower arm	foot-shaped, fused	subulate, separated	foot-shaped, fused
Upper arm length	ca. 5 mm	ca. 5 mm	ca. 3 mm
Lower arm length	ca. 1.5 mm	ca. 1.8 mm	ca. 1.5 mm
Nutlets	pale brown, ca. 2 mm	brown, ca. 1.5 mm	black, ca. 0.8 mm

## ﻿Taxonomy treatment

### ﻿ Salvia penghuana

Taxon classificationPlantaeLamialesLamiaceae

G.X.Hu & C.L.Xiang sp. nov.

1CC6C3B5-650D-5D8C-BFDD-265469C287E2

urn:lsid:ipni.org:names:77347687-1

Figs 1 , 2

#### Type.

China • Guizhou Province: Libo County, Yaoshan Town, Pobashao, karst forest margin, elevation 790 m, 1 May 2022, *G. X. Hu & Y. F. Geng 758* (holotype: GACP!; isotypes: GACP!, KUN!).

#### Diagnosis.

*Salviapenghuana* is similar to *S.filicifolia*, but differs in having 6-flowered verticillasters (vs. 6–10-flowered verticillasters), ovate-lanceolate bracts (vs. linear-lanceolate), purple corolla (vs. yellow or white), foot-shaped fused lower arms of connective (vs. subulate separated lower arm). It is also similar to *S.cavaleriei*, but differs by having 3–4-pinnate leaves (vs. simple to 2-pinnate), ovate-lanceolate bracts (vs. lanceolate), puberulent calyx (vs. glabrous), longer upper arms of connective (ca. 5 mm vs. ca. 3 mm).

**Figure 1.**
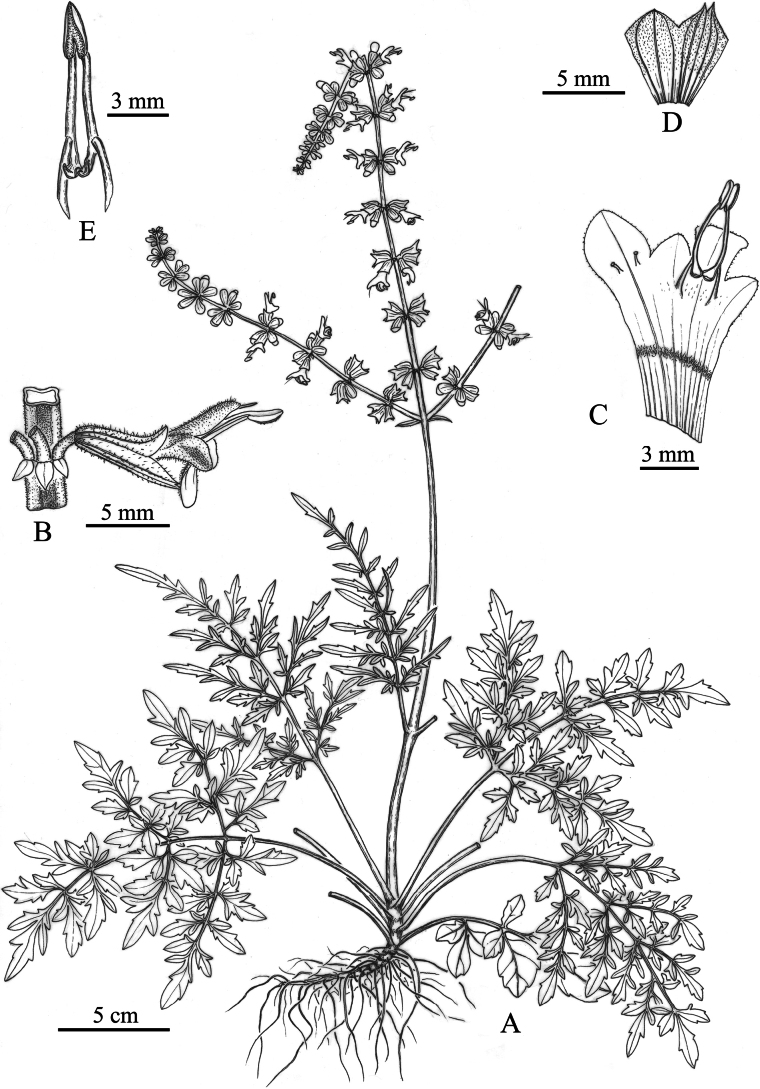
*Salviapenghuana* sp. nov. **A** habit **B** flower **C** dissected corolla **D** dissected calyx **E** fertile stamens. Drawn by Xiao-Yu Wang from the holotype.

#### Description.

Perennial herbs. Roots fibrous, 5–15 cm long. Stems erect, 10–60 cm tall, finely minutely pubescent or glabrous, simple, or branched from the base. Leaves mostly basal, cauline leaves absent or 1–2 paired; the most basal leaves 1–2-pinnate, usually 1-paired, caducous, petiole 4–10 cm long, terminal leaflets ovate, margin serrate; other basal leaves 3–4-pinnate, petiole 6–12 cm long, leaf blades ovate, 9–16 × 8–13 cm, lobes numerous, elliptic to linear-lanceolate, margin entire or few lobulate, terminal leaflets lanceolate, ca. 7 × 2 mm, adaxially dark green, glabrous or sparsely puberulent, abaxially greenish or purplish-brown, glabrous or villous along veins. Verticillasters 6-flowered, in racemes or panicles; rachis puberulent and glandular pilose. Bracts ovate to lanceolate, 4–6 × 1.5–2.5 mm, apex acuminate, margin entire, glabrous; bracteoles similar to bracts in shape but smaller. Pedicels 2–3 mm long, puberulent. Calyx tubular-campanulate, 4–6 mm long, bilabiate to one-fourth its length, pale purple, sparsely glandular or puberulent along veins outside, glabrous or apically fine strigose inside; upper lip semicircular-triangular, ca. 2 × 3 mm, margin entire; lower lip ca. 2 × 2.5 mm, shallowly 2-toothed, teeth triangular, apex acuminate. Corolla purple, 9–13 mm long, densely puberulent or glandular hairs; corolla tube 6–9 mm long, creamy yellow inside calyx tube, ca. 1 mm wide, densely puberulent annulate, gradually dilated after extending out of the calyx tube, purple, ca. 2 mm wide at the throat, sparsely villous; lips subequal, upper lip oblong, 3–4 × 2–3 mm, apex emarginated; lower lip 3-lobed, middle lobe subrectangular, 3–4 × 2.5–3.6 mm, lateral lobes oval-triangular. Fertile stamens 2, purple, glabrous, filament ca. 1.5 mm long; connective ca. 6.5 mm long, upper arm ca. 5 mm long, the lower arm ca. 1.5 mm long; anterior thecae oblong, ca. 1.5 mm long, fertile, connivent; posterior thecae boot-shaped, sterile, fused. Staminodes 2, 0.7–1.1 mm long. Style exerted slightly, stigmatic lobes unequal, posterior lobe shorter. Nutlets ellipsoid, pale brown, glabrous, ca. 2 mm long.

**Figure 2.**
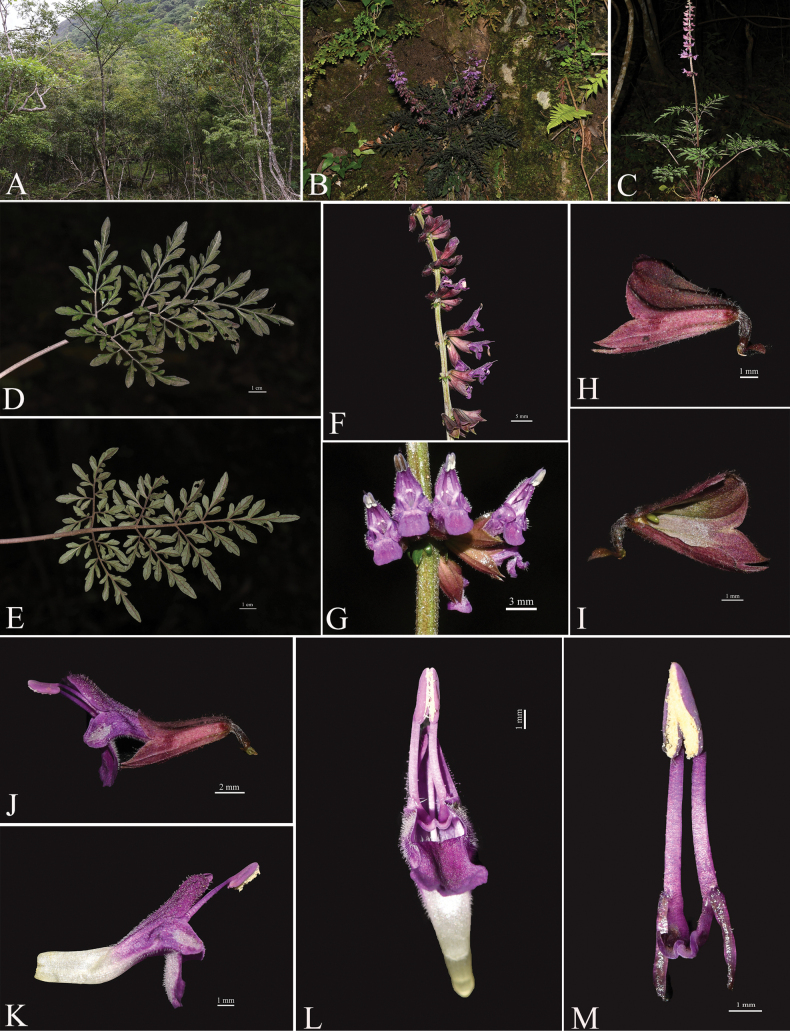
*Salviapenghuana* sp. nov. **A** habitat **B, C** plant **D** leaf (adaxial surface) **E** leaf (abaxial surface) **F** inflorescence **G** verticillaster **H** calyx (external view) **I** calyx (internal view) **J–K** corolla (side view) **L** (front view) **M** fertile stamens. Photographs by Guo-Xiong Hu.

#### Distribution and habitat.

**Figure 3.**
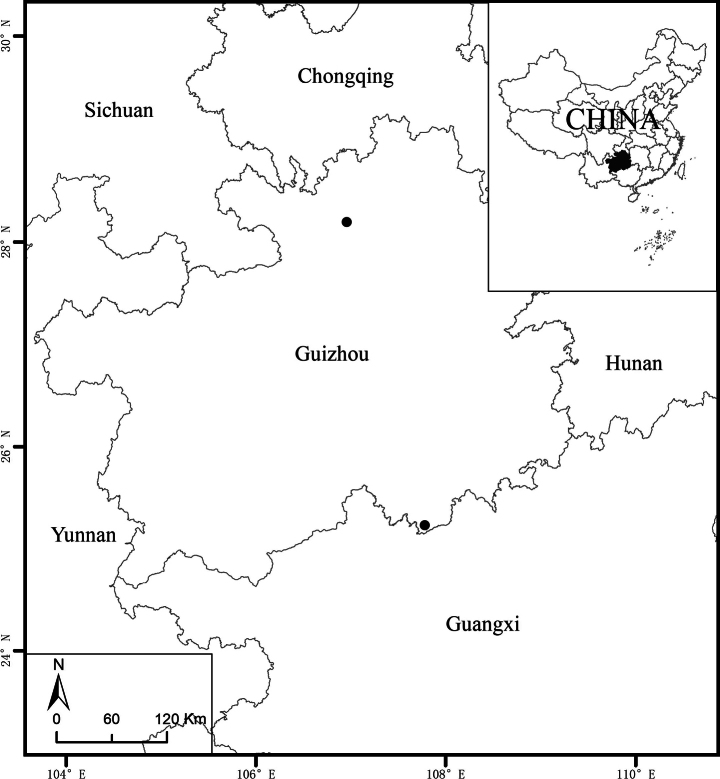
Known geographic distribution of *Salviapenghuana*.

The new species is currently only known from Libo and Suiyang counties, Guizhou Province, China, at elevations between 770 and 1220 m (Fig. 3). Both populations grow in karst evergreen and deciduous broad-leaved mixed forest. The common companion species include *Handeliodendronbodinieri* (H. Lév.) Rehder, *Sarcococcaruscifolia* Stapf, *Selaginellauncinata* (Desv.) Spring, Hederanepalensisvar.sinensis (Tobler) Rehder, *Asterageratoides* Turcz., and *Ajugadecumbens* Thunb.

#### Phenology.

Flowering from April to June, and fruiting from May to June.

#### Etymology.

The specific epithet ‘penghuana’ was selected to honor Prof. Hua Peng, who is a renowned expert on plant taxonomy at the Kunming Institute of Botany, Chinese Academy of Sciences, China.

#### Vernacular name.

Chinese Mandarin: Péng huá shǔ wěi cǎo (彭华鼠尾草)

#### Conservation status.

Currently, two populations are found in Guizhou, China. The population in Libo is close to Maolan National Nature Reserve, and another population in Suiyang is located in the Kuankuoshui National Nature Reserve. Two populations have no plausible threats, and the area is relatively well-known. Under IUCN criteria, the species was categorized as “Least Concern” (IUCN 2024).

#### Additional specimens examined

**(Paratypes).** China • Guizhou: Suiyang County, Kuankuo Town, Honghe village, elevation 1182 m, 4 May 2024. W. Wu & L. Chen *sy01* (GACP) • Guizhou Province: Libo County, Yaoshan Town, Pobashao, karst forest margin, elevation 790 m, 1 May 2023, G. X. Hu & W. Wu 779.

#### Notes.

Based on molecular and morphological evidences, Hu et al. (2018) established the subg. Glutinaria, of which eight sections were recognized. Sect. Sobiso (Raf.) G.X.Hu, A.Takano & B.T.Drew is characterized by fibril roots, small calyx (4–7 mm), small corolla (5–10 mm), and completely reduced posterior thecae. *Salviapenghuana* has these synapomorphies and therefore should be included in this section. Within sect. Sobiso, two lineages were recognized. The *Salviachinensis* group mainly consists of species distributed to China and a total of 17 species were reported (Hu et al. 2018; Wei et al. 2019). This group is characterized by the stamen movement whereby the upper connective arms bend downward from the upper lips at early anthesis to the middle lobe of the lower corolla lips at the end of flowering (Hu et al. 2018). This stamen movement is considered to be a diagnostic between the *S.chinensis* group and *S.lutescens* group endemic to Japan and Taiwan Island. A similar phenomenon is also observed in this new species, so *S.penghuana* should be placed in the *S.chinensis* group.

## Supplementary Material

XML Treatment for Salvia penghuana

## ﻿Additional information

### Author ORCIDs

Feng-Jin Qiu https://orcid.org/0009-0005-2909-7488

Deng-Li Yu https://orcid.org/0009-0005-3206-6383

Hong-Bo Lan https://orcid.org/0009-0007-1715-7687

Ming-Tai An https://orcid.org/0000-0003-3886-0287

Yan-Fei Geng https://orcid.org/0009-0005-4160-4497

Chun-Lei Xiang https://orcid.org/0000-0001-8775-6967

Guo-Xiong Hu https://orcid.org/0009-0007-6983-0626

### Data availability

All of the data that support the findings of this study are available in the main text.
